# Studies on the Authorship of Albumen Vintage Photographs: A Combined Experimental and Chemometric Approach

**DOI:** 10.3390/molecules29102170

**Published:** 2024-05-07

**Authors:** Monika Adamowska, Izabela Zając, Marek Grzegorz Sawicki, Wojciech Hyk

**Affiliations:** 1 Faculty of Chemistry, University of Warsaw, Pasteura 1, PL-02-093 Warsaw, Poland; m.adamowska2@student.uw.edu.pl (M.A.); m.sawicki@chem.uw.edu.pl (M.G.S.); 2 Faculty of Conservation and Restoration of Works of Art, Academy of Fine Arts in Warsaw, Wybrzeże Kościuszkowskie 37, PL-00-379 Warsaw, Poland; izabela.zajac@gmail.com; 3 Faculty of Chemistry, Biological and Chemical Research Center, University of Warsaw, Żwirki i Wigury 101, PL-02-089 Warsaw, Poland

**Keywords:** albumen photographs, energy dispersive X-ray fluorescence, Fourier transform infrared spectroscopy, principal component analysis

## Abstract

The differences in albumen photographs from vintage photographic studios were identified by energy-dispersive X-ray fluorescence spectroscopy and Fourier transform infrared spectroscopy. The results inspired the concept of finding common features characteristic of a given photographic studio. The obtained measurement data (i.e., positions of vibrational bands for characteristic groups of albumen and the mass contents of chosen elements) were analyzed chemometrically by employing the Principal Component Analysis (PCA). The PCA technique allowed us to reduce the number of relevant experimental parameters characterizing the unique features of the photographic objects. The two major components were able to distinguish the photographic objects in terms of their authorship and the time to produce a photograph. The method developed was examined for a selected group of photographs consisting of albumen prints from three Polish photographic ateliers. To validate ED-XRF measurements and, consequently, the chemometric findings, reference albumen photo samples were designed and prepared. The empirical functional relationships between the content of photochemically reduced silver particles on the photographic paper and several physicochemical factors, including time of exposure to UV light, AgNO_3_ concentration in a fixed bath, and concentrations of other additives, were proposed. These results can be used for the prediction of the experimental conditions under which the investigated photographs were developed.

## 1. Introduction

The albumen technique was the most popular process of printing positives on paper in the 19th century. For this reason, albumen images are an important part of historical collections of photographs. The way the albumen photographs were prepared varied depending on the procedure employed in a photographic studio. This is very important because nineteenth-century photographic studios, based on the same known rules [1,2], developed individual formulas for preparing an albumen photograph [3]. Usually, those formulas were kept secret, as they were the success of the studio and the photographer. This resulted in differences in the structure and composition of albumen. Therefore, it was assumed that based on physicochemical features that were specific to the products of a given photographic studio, it would be possible to determine the authorship or probable provenance of photographs without knowing signatures, the company’s marking of the photographic studio, and the stylistic criteria used in a given photographic studio (i.e., decorations, furniture, and backgrounds). Instead of the above factors, it was decided to analyze the method of taking pictures from the selected studios.

Initially, albumen photographic paper was made in a photoatelier. In 1854, the first commercially produced albumen photographic paper appeared on the market. However, it was only coated with salted albumen and had to be sensitized with a silver nitrate solution. Since 1872, albumen photographic paper with a longer shelf life has been available to the market. Despite this, many photographers did not trust the purchased product for economic reasons, so they preferred to make the paper themselves. Then, the differences between the formulas and the method of making photographic prints began at the stage of choosing the right paper for the base of the photo. Of course, recommendations were made in this regard, which appeared in guides and specialist press [1,2]. However, it was the photographer or the studio who first had to examine the suitability of the selected components and how to prepare them (e.g., select the sizing material along with the sizing procedure) and, similarly, they had to choose the paper product or products available in a given geographical area.

Therefore, one might expect some variations between the individual studios due to their unique procedures for preparing photographs. This might result in differences in the structure, composition, and mechanical properties of albumen layers. The following factors may account for the discrepancies: the conditions under which albumen was prepared, the method of its application, and a variety of chemical additives (for example, sodium chloride or ammonium chloride). In addition to this, each photographer or workshop prepared their own mixture of photosensitive substances. The specific selection of individual components, as well as the proportions between them, led to the creation of distinctive features [4,5,6,7,8].

Also, the quality of water used in the photographic studio was an important factor. Rainwater was recommended primarily, although its chemical composition could vary depending on the degree of industrialization of the site and the possible presence of gaseous pollutants (i.e., sulfur dioxide, nitric oxide, or hydrogen sulfide) and certain amounts of mineral salts or other additives.

The majority of the above-described factors (variables) can be quantified by employing analytical techniques, such as Fourier transform infrared spectroscopy (FTIR) and X-ray Fluorescence Spectroscopy (XRF) [9,10,11,12,13,14]. These instrumental techniques were, for instance, successfully employed for the non-destructive [15,16,17] identification of photographs, paintings, and other objects of cultural heritage [12,13,14,15,16,17,18,19,20,21,22,23]. The energy dose provided by X-ray radiation and utilized by the ED-XRF spectrometer had no noticeable effect on old photographs and paintings if measurements were performed occasionally [24,25]. The variability among the measurement results and possible correlations between the measured quantities can be studied conveniently by means of chemometric methods [26]. The Principal Component Analysis (PCA) was employed to reduce the number of relevant variables in the detailed analysis of a selected group of objects, consisting of albumen prints from the photographic studios of Beyer [27], Brandel, and Mieczkowski.

To test the performance of the ED-XRF technique employed for the analysis of the silver content in photographic objects, reference photo samples for the albumen technique were designed and prepared. The production of the reference materials was based on well-documented procedures. The produced materials were then characterized by ED-XRF and FTIR techniques. The empirical functional relationships between the content of photochemically reduced silver particles on the photographic paper and several physicochemical factors, including time of exposure to UV light, silver nitrate concentration in a fixed bath, and concentrations of other additives, were proposed. These results can be used for the prediction of experimental conditions under which the photographs were developed.

## 2. Results

### 2.1. Theoretical Basis of Principal Component Analysis (PCA)

The essence of Principal Component Analysis [28] consists of determining the components (factors) in the form of linear combinations of measured quantities that characterize the studied objects. The measured quantities (i.e., element contents and IR wavenumbers) constitute a set of the initial variables. The PCA method allows one to indicate the main components, i.e., those that explain the largest part of the initial variability (variance) among the variables studied. The main component (in which the variance is maximized) is then the feature that characterizes the phenomenon studied most. An additional analysis of the main components also makes it possible to indicate the initial variables that have the largest contribution to the established main components. The other components are mutually uncorrelated (orthogonal to each other) and defined in such a way as to maximize variability (variance), which was not explained by the previous components. The PCA technique reduces the number of initial variables to describe the phenomenon being studied. In addition to this, it detects regularities (relations) between variables, verifies them, and classifies objects in the new coordinate system defined by the calculated principle components.

The PCA calculation procedure includes the following steps (assuming *n* objects characterized by *m* variables (*x*)):

1. The calculation of arithmetic mean values (*x_i_*) and standard deviations (*SD*(*x_i_*)) of individual variables (either metal concentrations or IR wavenumbers, *i* = 1, …, *m*, *m*—the number of variables, with each variable represented by n measurements, *n*—number of samples/objects).
2. Data scaling, especially in the case of the incomparability of initial data, i.e., in the case when the initial variables represent different measured values (measurements in different units) or in the case of large differences in the values of the measured quantity).

(1) $$X_{ij}=\frac{x_{ij}-x_{i}}{SD\left(x_{i}\right)}$$

where *x_ij_*—*j*-th measurement of the *i*-th variable (*i* = 1, …, *m*, *j* = 1, …, *n*), *x_i_*—the mean value of the *i*-th variable (averaged value of *n* measurements), and *SD*(*x_i_*)—the standard deviation of the *i*-th variable.

3. The construction of the data matrix **X: X = [X_1_, X_2_, …, X_m_]**—matrix size *m* × *n*.
4. The construction of correlation matrix **R** (matrix formed from correlation coefficients between particular pairs of variables *r*(**X_i_**, **X_k_**) for *i*, *k* = 1, …, *m* (*r*(**X_i_**, **X_i_**) = 1). **R = [*r*(X_1_), *r*(X_2_), …, *r*(X_m_)]**—matrix size *m* × *m*.
5. Searching for the eigenvalues of the correlation matrix **R**, i.e., *λ*_1_, *λ*_2_, …, *λ_m_*.
6. Determining the eigenvectors of the correlation matrix **R**, i.e., **v**_1_, **v**_2_, …, **v***_m_*, where the *i*-th vector can be represented by the following notation.

(2) $$v_{i}=\left[\begin{array}{c} a_{i1}\\ a_{i2}\\ \ldots \\ a_{im} \end{array}\right]$$

Elements of the *i*-th eigenvector of the correlation matrix are the coefficients of the *i*-th component *Z_i_* (*i* = 1, …, *p*, *p* ≤ *m*). They define the form of the linear combination of the *i*-th component, i.e.,

(3) $$Z_{i}=a_{i1}X_{1}+a_{i2}X_{2}+\cdots +a_{im}X_{m}$$

7. Determination of the contribution of the variance of the i-th Z component in the total variance based on the eigenvalues of the correlation matrix R.

(4) $$\%Var\left(Z_{i}\right)=\frac{\lambda_{i}}{\lambda_{1}+\lambda_{2}+\cdots +\lambda_{m}}\times 100$$

(5) $$SD\left(Z_{i}\right)=\sqrt{\lambda_{i}}$$

8. Selection of the main components based on the analysis of the scree plot. The factor of scree is given by eigenvalues (and consequently Zi components) that have the smallest contribution to the sum of eigenvalues (i.e., those components that, together, explain less than 10–15% of the total variance of the initial variables).
9. The determination of correlation coefficients between variables and their main components (the correlation coefficient between the vector of the main component Z and the vector of variable *X_i_*, *r*(Z, *X_i_*)).
  These calculations are illustrated by a pie chart of variables in the coordination system of the main components. Correlation coefficients act as the coordinates of points representing particular variables in the main component graph. The length of the variable vector represents the strength of the correlation and its direction—the sign of the correlation coefficient.
10. The calculation of determination coefficients (squares of correlation coefficients between variables and main components). The values of determination coefficients determine the degree of representation of a given component by particular variables quantitatively.
11. The graphical location of individual objects in the coordinate system of main components (usually Z1 and Z2). The coordinates of a given object (z1, z2) are determined from the expressions of the Z1 and Z2 components (obtained in point 6.). The calculations are illustrated by a graph in the coordinate system of the main components.

All numerical calculations and graphical presentations were performed in the R environment.

### 2.2. The Characterization of Reference Albumen Photo Samples

The reference materials reproduce the content of metallic silver particles generated by the photochemical reaction and initiated by UV light illumination. The XRF quantification of the silver content in the prepared albumen photo samples was related to the known total concentration of silver absorbed by albumen paper before UV light exposure. The XRF quantification was performed via the calibration program from the standard library built into the operating system of the handheld XRF spectrometer. The combined uncertainty of the XRF determinations was based on the reproducibility of the measurement results. The representative samples employed for the experiments are shown in Figure S1 (Supplementary Material).

#### 2.2.1. The Effect of the AgNO_3_ Concentration and UV Illumination Time on the Content of Silver Particles Determined in the Photo Samples

The empirical relations between the concentration of the nitrate salt of silver (I) (*c_AgNO3_*) in the fixed bath, the exposure time to UV light (*t*), and the resulting XRF content of metallic silver particles (*x_Ag_* (XRF)) accumulated in photo paper are presented graphically in Figure S2 (Supplementary Material). As predicted, the content of silver in the developed materials increases monotonically with the increase in both the AgNO_3_ concentration and the exposure time. It is also seen that the magnitude of the *x_Ag_* (XRF) parameter remains unchanged for the UV illumination time no longer than 120 s. The exposure time of 120 s can, therefore, be treated as the changing point in the empirical relations presented in Figure S2.

**Figure 1:**
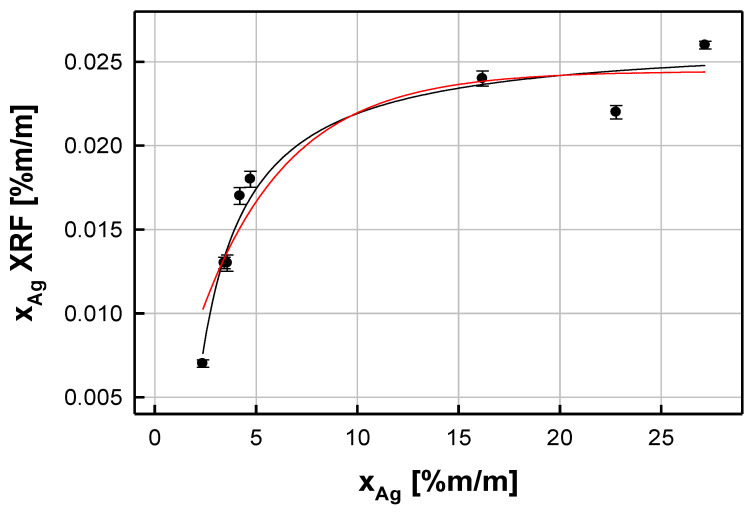
Dependence between the resulting XRF content of metallic silver particles (*x_Ag_* (XRF)) and the silver (I) content (*x_Ag_*) absorbed by the photographic paper. The experimental points are associated with standard uncertainties.

The next step in the evaluation of the prepared reference photo samples was related to establishing the empirical model represented by the functional dependence between the XRF quantification of the silver content (*x_Ag_* (XRF)) in the prepared albumen photo samples and the known total concentration of silver (*x_Ag_*) absorbed by albumen paper before UV light exposure. The *x_Ag_* (XRF) parameter was correlated with the silver (I) content (*x_Ag_*) absorbed by the photographic paper and used for the production of the reference material. The nature of the studied phenomenon (represented by a relationship between the adsorbate (Ag^+^) content in photo samples soaked with the aqueous solution of adsorbate (Ag^+^) and the content of the adsorbate (Ag) adsorbed on the surface of the paper under the equilibrium conditions) suggested the use of the exponential model to describe the trend among the experimental points. The resulting dependence obtained for the selected UV exposure time (600 s) is presented in Figure 1. It can be represented by the following regression equation:

(6) $$x_{Ag}\left(\mathrm{XRF}\right)=a\left(1-e^{-bx_{Ag}}\right)\;\;$$

where *a* ± *SD*(*a*) = 0.0244 ± 0.0013, *b* ± *SD*(*b*) = (0.229 ± 0.033) % (m/m) (*SD*(*a*) and *SD*(*b*) represent standard deviations of the regression coefficients *a* and *b*, respectively.

The *x_Ag_* determination required the knowledge of the volume (*V*) of the AgNO_3_ aqueous solution of the fixed bath absorbed by a piece of photographic paper and the change in the AgNO_3_ concentration caused by the absorption. The *V* parameter, which reflects the solution uptake by the piece of photo paper, can be easily determined by measuring the loss of the solution mass (with known density) in the fixed bath. The presence of the photo paper in the fixed bath did not change AgNO_3_ concentration. This implies that the photo paper samples do not accumulate specifically with the Ag^+^ species. The Ag^+^ content before and after the absorption experiment was quantified using the dedicated cuvette test (Hach, DR3900, Ames, IA, USA) and was confirmed by ICP-MS (Perkin Elmer, Nexion 2000, Waltham, MA, USA) analysis. Therefore, the *x_Ag_* quantity can be expressed as follows:

(7) $$x_{Ag}=\frac{m_{Ag}}{m_{p}}=\frac{c_{{\mathrm{AgNO}}_{3}}\times V\times M_{\mathrm{Ag}}}{m_{p}\times M_{{\mathrm{AgNO}}_{3}}}$$

where *M*_Ag_ and *M*_AgNO3_ are the molar masses of Ag and AgNO_3_, respectively, *m_p_* is the mass of the piece of photographic paper, and *c*_AgNO3_ represents the concentration of AgNO_3_ in the fixed bath.

#### 2.2.2. The Effect of the AgNO_3_ Concentration and UV Illumination Time on the Position of the Characteristic IR Signals of Albumen Layers

**Figure 2:**
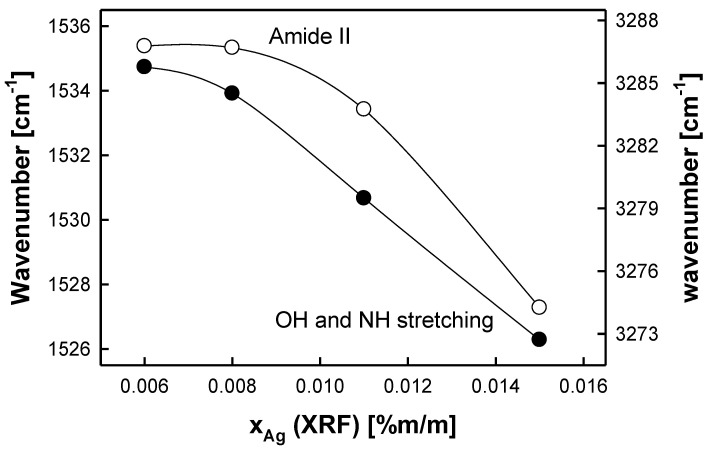
Dependence between the resulting XRF content of metallic silver particles (*x_Ag_* (XRF)) and the position of characteristic IR signals of the albumen layer (NH and OH stretching bands (black circles, right axis), amide II NH bending and CN stretching bands (white circles, left axis)).

There are three characteristic IR signals observed for albumen. These are as follows: NH and OH stretching bands (3250–3550 cm^−1^), amide I NH bending and CO stretching bands (1600–1690 cm^−1^), and amide II NH bending and CN stretching bands (1480–1580 cm^−1^). It was found that the positions of amide II and NH/OH IR signals are inversely proportional to the measured quantity reproduced by the reference samples (*x_Ag_* (XRF)), while the position of the IR signal corresponding to amide I reveals no tendency with respect to the quantity of *x_Ag_* (XRF) quantity. The results are given in Figure 2. The changes in the wavenumbers corresponding to the peaks for amide II and NH/OH stretching bands are significantly greater than the magnitude of the random error associated with the IR measurements. Each point included in Figure 2 represents the average value (from at least 16 replicates), with the coefficient of variation being less than 0.08%. The results referring to amide I are not shown.

The “strength” of these dependencies can be quantified by the following average sensitivity coefficients (i.e., the slopes of the linear parts of the empirical dependencies presented in Figure 2), which are −168 and −360 cm^−1^/(%Ag (m/m)) for amide II and NH/OH stretching bands, respectively. Thus, the IR analysis of albumen photographs allows one to indirectly determine the silver content present in the photographic paper.

### 2.3. Chemometric Analysis of XRF Determinations for Vintage Photographs

**Figure 3:**
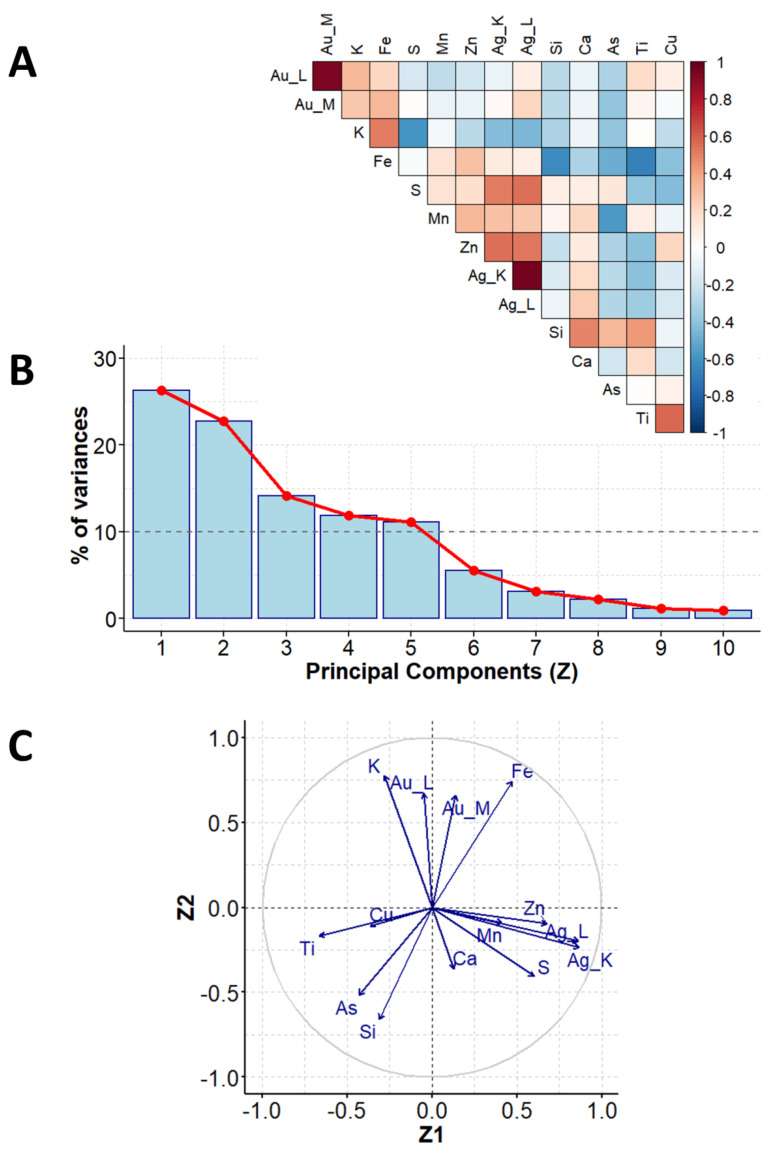
PCA results for XRF measurements: correlation coefficients for all unique pairs of variables (**A**), scree plot, showing contributions of individual components to the total variance (**B**), and correlation between the main components no. 1 and 2 (Z1 and Z2) and variables (**C**).

**Figure 4:**
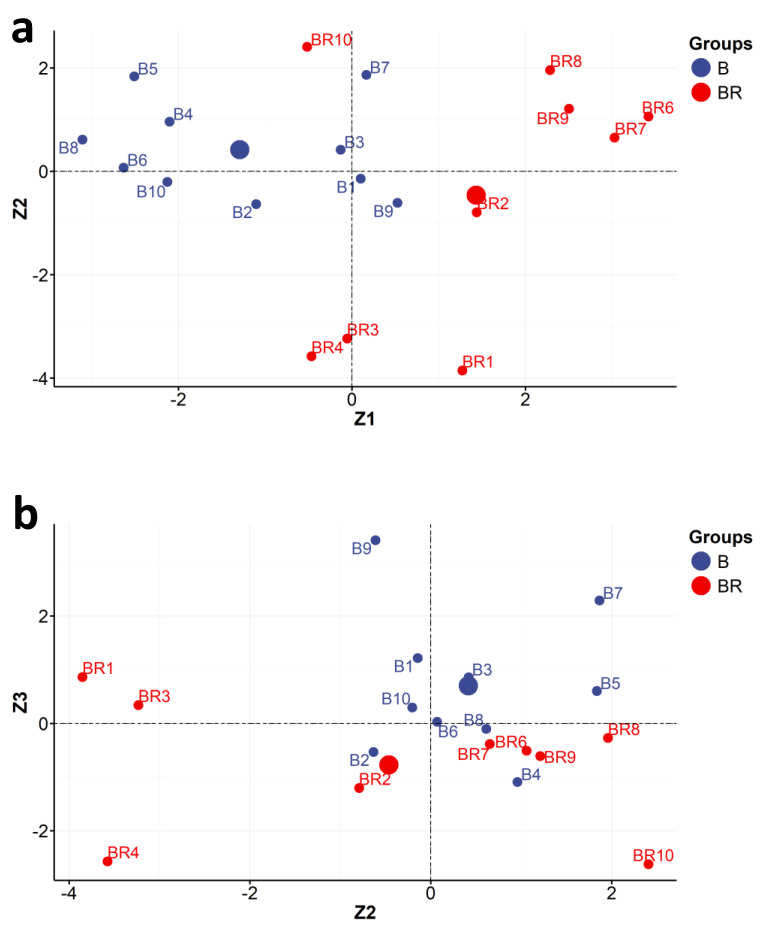
Locations of individual objects (studios: Brandel (BR) and Beyer (B)) in the coordination systems defined by the main components no. 1 and 2 (Z1 and Z2) (**a**) and no. 2 and 3 (Z2 and Z3) (**b**) (XRF measurements).

A representative XRF spectrum with spectral assignations recorded for the selected vintage photo (B1) is presented in Figure S3 (Supplementary Material). The first step of PCA was to establish the data matrix. The XRF instrumental responses (counts) for elements (Si, S, K, Ca, Ti, Mn, Fe, Cu, Zn, As, Ag (K line), Ag (L line), Au (L line), Au (M line)) were treated as experimental variables and formed columns of the data matrix. Each row of this matrix corresponded to the object investigated, i.e., the photograph belonging to either the Brandel or Beyer studio. The data matrix was evaluated according to the procedure demonstrated in Section 2.1, and the obtained PCA results (Figure 3 and Figure 4) are given as follows:

- A diagram of correlation coefficients for all unique pairs of variables (Figure 3A, step 4 in Section 2.1);
- The scree plot representing the contributions of individual components to the total variance (Figure 3B, step 7 in Section 2.1);
- A pie chart demonstrating the correlation between the main components no. 1 and 2 (Z1 and Z2) and variables (Figure 3C, step 9 in Section 2.1);
- A plot of the locations of individual objects in the coordination systems defined by the main components no. 1 and 2 (Z1 and Z2) and no. 2 and 3 (Z2 and Z3) (Figure 4a,b, step 11 in Section 2.1); these coordinates are the scores returned by the PCA analysis.

### 2.4. Chemometric Analysis of FTIR Measurements for Vintage Photographs

**Figure 5:**
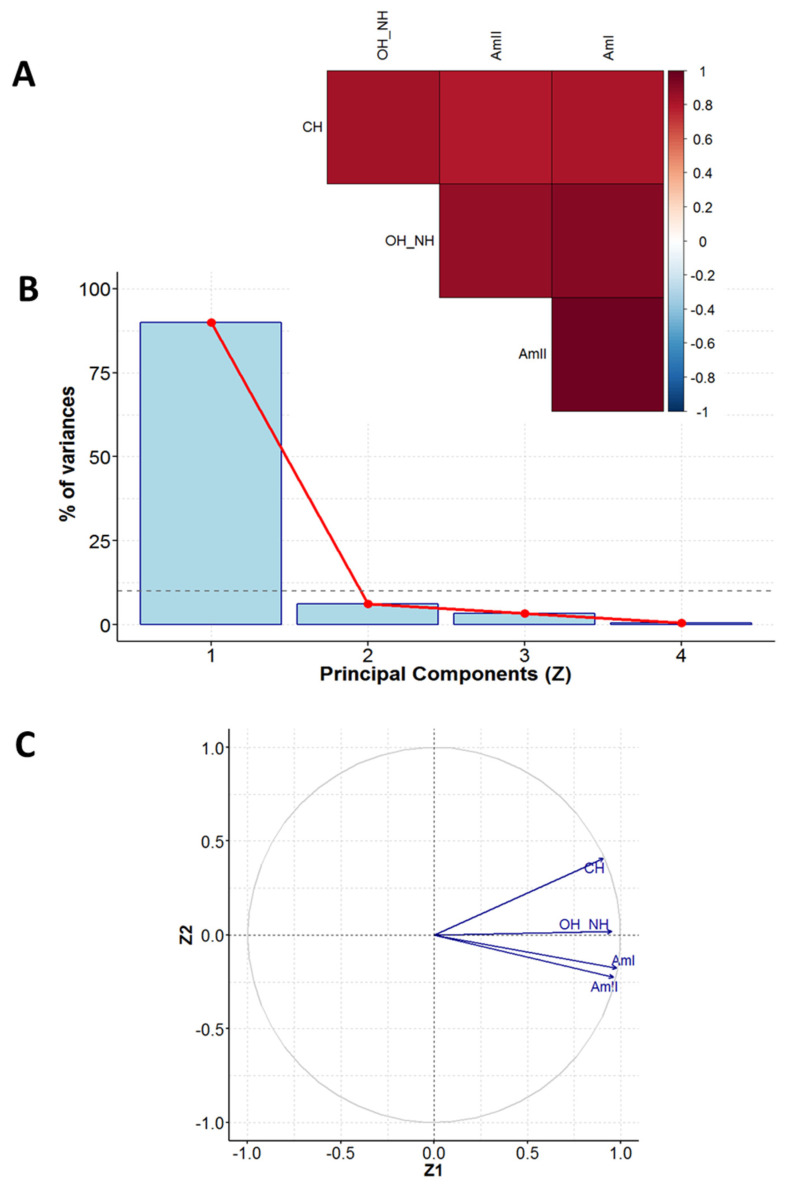
PCA results for FTIR measurements: the correlation coefficients for all unique pairs of variables (**A**), scree plot, showing contributions of individual components to the total variance (**B**), and correlation between the main components no. 1 and 2 (Z1 and Z2) and variables (**C**).

**Figure 6:**
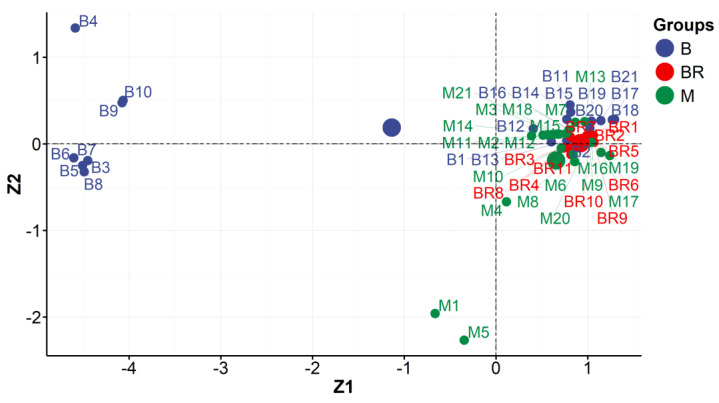
Locations of individual objects (studios: Brandel (BR), Beyer (B), and Mieczkowski (M)) in the coordination system defined by the main components no. 1 and 2 (Z1 and Z2) (FTIR measurements).

A representative IR spectrum recorded for the selected vintage photograph (BR11) marked with symbols for characteristic frequencies is presented in Figure S4. Qualitatively, the IR spectra of all vintage albumen photographs studied looked similar. The characteristic frequencies of stretching vibrations observed for albumen in IR signals were treated as experimental variables and formed columns of the data matrix. The following signals were chosen: the amide I signal (NH bending and CO stretching bands, labeled as “AmI”), amide II (NH bending and CN stretching bands, labeled as “AmII”), CH stretching band (labeled as “CH”), and NH and OH stretching bands (labeled as “OH_NH”). Each row of the matrix corresponds to the object investigated, i.e., the photograph belonging to a specific studio. The following PCA results (Figure 5 and Figure 6) were obtained by employing the procedure demonstrated in Section 2.1:

- A diagram of correlation coefficients for all unique pairs of variables (Figure 5A);
- The scree plot representing the contributions of individual components to the total variance (Figure 5B);
- A pie chart of the correlation between the main components no. 1 and 2 (Z1 and Z2) and variables (Figure 5C);
- A plot of the locations of individual objects in the coordination system defined by the main components no. 1 and 2 (Z1 and Z2) (Figure 6); these coordinates are the scores returned by the PCA analysis.

## 3. Discussion

### 3.1. XRF Measurements

Variability among the XRF element determination results can be reduced to five dimensions (these five main components explain more than 82% of the initial variability among the measurement data). The description is limited to the three main components (Z1, Z2, Z3) accounting for 61.5% of the total initial variance (Figure 3B). 

According to the results presented in Figure 3C, the Z1 main component is significantly correlated (with correlation coefficients greater than or equal to 0.7) to the contents of Ag (positive correlation), Zn (positive correlation), and Ti (negative correlation). The Z2 component is significantly correlated with the contents of K (positive correlation), Au (positive correlation), and Si (negative correlation).

The main Z1 component, to some extent, differentiates the objects of Beyer and Brandel. Based on the analysis of the location of individual objects in the space delimited by the main components, Z1 and Z2, it can be seen that the photographs by K. Brandel are characterized by a higher content of silver and zinc in relation to the photographs of K. Beyer.

It should be emphasized that the main components, Z1 and Z2, divided the objects into several distinct groups (Figure 4a). The Z1 component makes the basic division. On its left side were mainly photographs of Beyer (B) marked with the following symbols: B2, B4, B5, B6, B8, B10. In turn, on the right side of the Z1 component, there were mainly Brandel’s photographs (BR): BR1, BR2, BR6, BR7, BR8, BR9. The Z1 and Z2 components are expressed by the following linear combinations of the measured parameters:

$$Z1=-0.3114X_{Si}+0.6010X_{S}-0.2802X_{K}+0.1292X_{Ca}-0.6638X_{Ti}+\;0.4104X_{Mn}+0.4697X_{Fe}-0.3653X_{Cu}+0.6771X_{Zn}-0.4325X_{As}+\;0.8673X_{AgK}+0.8612X_{AgL}-0.0483X_{AuL}+0.1387X_{AuM},$$

and

$$Z2=-0.6591X_{Si}-0.4076X_{S}+0.7747X_{K}-0.3612X_{Ca}-0.1672X_{Ti}-\;0.0915X_{Mn}+0.7418X_{Fe}-0.1070X_{Cu}-0.0955X_{Zn}-0.5167X_{As}-\;0.2356X_{AgK}-0.1985X_{AgL}+0.6709X_{AuL}+0.6596X_{AuM},$$

These model equations allow one to predict the location of a photo object in the Z1, Z2 coordination system and, consequently, to sort objects by their provenance. 

Additionally, considering the distribution of objects between the Z1 and Z2 axes, it is possible to distinguish four clusters of the objects examined (four groups).

Group I comprises five objects: B4, B5, B6, B8, B10. Most of them, apart from B10 (Figure 3 and Figure 4a: quarter Z1 < 0 and Z2 > 0), have a positive correlation with potassium (K) and gold (Au). They all, except object B10, come from a single copy of the Karol Beyer publishing photo album PPA2 entitled “Views of Warsaw”/“Widoki Warszawy” from the National Museum in Warsaw [MNW DI 105371 (1–24)]. These photos were stuck on paper cards (plaques) decorated with an identical motif and taken in the years 1858–1859.

The object B10 (quarter Z1 < 0 and Z2 < 0) has a correlation with silicon (Si) and arsenic (As). These elements were found in this paper. The intriguing presence of arsenic can be explained by the admixture of cobalt smalt, which was supposed to prevent the yellowing of the paper. Arsenic is also a component of disinfectants and could theoretically also be used as an addition to the glue when combining photographs with a paper card with cardboard. Object B10 is an example of carte de visite (CV) photography by Karol Beyer but comes from another album, namely from the classic family photo album CFPA1 [MHK Fs4452/IX/48]. Classic family photo albums were available commercially as empty volumes with specially prepared pages, in which windows were cut to contain photographs of a specified size (in this case, carte de visite photography at c. 6 cm × 9 cm was identified on cardboard with lithographic decoration around the photograph and on its back; this can be compared with Figure S5B). This left the owner with the possibility of placing and choosing the order and hierarchy of images in a CFPA1 album.

In group II, there were four photos, B1, B3, B7, and B9, taken by Beyer (Figure 4a: along Z2). They form a very interesting mixed group. Most of them (except B9) come from two editions of Beyer’s publishing photo album entitled “Views of Warsaw”/“Widoki Warszawy”. Object B1 comes from the publishing album PPA1 [MW, AF 9773], and objects B3 and B7 come from album PPA2 [MNW DI 105371 (1–24)]. Importantly, both albums came from two different collections, namely PPA1 from the collection of the Museum of Warsaw (MNW) and PPA2—from the Museum of Warsaw (MW). Object B9 is an example of carte de visite (CV) photography by Karol Beyer and comes from the classic family photo album CFPA1 [MHK Fs 4452/IX/48].

In group III (Figure 3 and Figure 4a: quarter Z1 > 0 and Z2 > 0), where correlations with iron (Fe) and gold (Au) appear, there are four objects (BR6, BR7, BR8, BR9). They form a very homogeneous group (compared with Figure S5A). All objects in this group came from the same publishing album made by Konrad Brandel PPA4 (“Views of Warsaw”/“Widoki Warszawy” MW Al.79, edition in red canvas).

In group IV, there were three photos: BR1, BR3, and BR4. These also formed a very homogeneous group of objects from the same Konrad Brandel’s publishing photo album PPA3 entitled the “Views of Warsaw”/“Widoki Warszawy” edition in brown canvas [MW AL. 78]. It can be seen that the BR1 object is located in the Z1 > 0 and Z2 < 0 quarter (Figure 4a), where positive correlation with zinc (Zn), silver (Ag), manganese (Mn), sulfur (S), and calcium (Ca) occurs. The BR3 and BR4 objects are located in the Z1 < 0 and Z2 < 0 quarter (Figure 4a), where correlations with silicon (Si) and arsenic (As) are observed.

Three objects (B2, BR2, and BR10) remained unclassified. The B2 photograph came from the publishing photo album PPA1 made by Karol Beyer entitled “Views of Warsaw”/“Widoki Warszawy” [MW AF 9766]. The BR2 object came from the publishing photo album PPA3 by Konrad Brandel entitled “Views of Warsaw”/”Widoki Warszawy” (brown canvas binding edition) [MW AL. 78]. The BR 10 object came from the publishing photo album made by Konrad Brandel PPA4 (“Views of Warsaw”/“Widoki Warszawy” MW Al.79, edition in red canvas). 

With the main components, Z2 and Z3 (Figure 4b), the objects of Brandel and Beyer are difficult to distinguish.

### 3.2. FTIR Measurements

Variability among the values of wavenumbers of characteristic bands for albumen (denoted by AmI, AmII, CH, and OH_NH) can be reduced to one dimension. The main component (Z1) explains almost 90% of the initial variability among the measurement data (Figure 5B). The Z1 component is significantly correlated with the values of wavenumbers for all considered groups (Figure 5C).

Figure 6 reveals that the main components Z1 and Z2 divided Beyer’s photography into two subgroups (group I: B3–B10 and group II: B1, B2, B11–B21). The majority of the photographs studied were not differentiated by the main factor. In terms of IR’s characterization, they can be considered homogeneous. However, considering the distribution of objects between the Z1 and Z2 axes, one may distinguish two clusters of the objects examined (two groups).

Group I. The great majority of Beyer’s isolated photographs (B3–B8) came from the same edition as the album PPA2, “Views of Warsaw”, which was prepared by Karol Beyer in 1858/1859 [MNW DI 105371 (1–24)]. The other two objects are photographs in formal format (B9 and B10), which come from the classic family album CFPA1 [MHK Fs 4452/IX] and date from the 1860s. Hypothetically, the main component of Z1 was used to extract objects taken at the same time and separate photos with a similar degree of degradation in the albumen layer. The latter conclusion is fully supported by IR measurement results obtained for the reference samples (Figure 2). This concept is confirmed by the fact that other objects by Beyer were (B1, B2) separated from group I and belonged to group II. They came from 1858/59 but from another edition of the album PPA1, “Views of Warsaw”, from the collections of the Museum of Warsaw [MW AF 9766], so they could be stored in other conditions and, thus, have different levels of degradation. 

In addition, the photographic studio prepared a larger amount of albumen and subjected it to fermentation processes (lasting about 3 months). Only then was a photo emulsion formed from it. At that time, the photographer took the necessary amount of albumen and left the rest for later use. During this time, unused albumen continued to ferment. So, hypothetically, differences in the extent of albumen degradation will be related to the use of albumen with varying degrees of fermentation and the subsequent production/preparation of prints for the next edition of the album.

Group II includes Beyer’s photographs (B1, B2—“Views of Warsaw” 1858/59 [MW AF 9766]. In addition, the group included Brandel objects (BR1—BR11). This group also included Mieczkowski’s photographs.

## 4. Materials and Methods

### 4.1. Preparation of the Reference Photo Samples

#### 4.1.1. Chemicals

The basic characteristics of chemicals used in our studies are presented in Table 1.

#### 4.1.2. Experimental Procedure

To prepare an albumen photograph, it is necessary to have a gelatine-covered paper that acts as a base surface for albumen. At first, 3.5% bovine gelatine powder was mixed with water and 1 g of aluminum potassium sulfate per 100 mL of water, which was then warmed up to a temperature range of 60–70 °C. Gelatine was poured into a container large enough not to fold or bend the prepared paper. Whatman blotting paper 1 was placed on the top of the gelatine surface, forming an even layer. Only one side of the paper was covered by gelatine in order to glue the other side to the backing in a later process. The gelatine-covered paper was left for a few days under the press to dry.

The dry gelatine-covered paper was later placed on albumen containing 3% (m/m) ammonium chloride by dropping it from a small height (about 1–2 cm). It is important to place the paper in a way that prevents the formation of air bubbles between the paper and the albumen. Also, albumen could not overflow onto the other side of the paper (side without gelatine). After about 15 min (the time needed for the paper to relax), the sheet was taken out with plastic tweezers. Excess albumen solution was drained off. The paper was hung on plastic clips and left overnight to dry out.

The prepared albumen paper was then immersed in a 50 mL AgNO_3_ solution with citric acid for 1 min. The following compositions of the solutions were employed in the following experiments: 1% AgNO_3_ with 0.5% citric acid; 1.5% AgNO_3_ with 0.75% citric acid; 2.5% AgNO_3_ with 1.25% citric acid; 5% AgNO_3_ with 2.5% citric acid; and 10% AgNO_3_ with 5% citric acid.

There was an attempt to apply a solution of silver nitrate with a brush in order not to waste so much solution, but it resulted in albumen layer damage.

Paper soaked in AgNO_3_ solution was hung on clips in a darkroom and left for about 3 to 5 h to dry (the time depended on the sheet size used). Dry sheets were then placed under a UV lamp and exposed for a chosen time (30 s/60 s/90 s/120 s/600 s). Then, the paper was immersed in eight baths (5 min each). This process involved the following sequence of chemical baths: 3% citric acid and 3% ammonium chloride solution (1:1 ratio); distilled water; 10% sodium thiosulfate; and 15 washes with distilled water.

The finished albumen photograph was later left on absorption paper to dry and was ready for XRF and IR analyses.

### 4.2. Instrumental Techniques

Energy dispersive X-ray fluorescence spectrometers (model Bruker Tracer III-SD, Bruker, and model Bruker Titan S1, Bruker, Middlesex, MA, USA) were used to determine the elemental contents in photographic objects. In the case of reference samples, at least 10 spots randomly selected on the surface of the material examined were analyzed. The obtained measurement results were then averaged, and standard uncertainty was evaluated. In the case of albumen photographs, XRF spectra were recorded at the dark area of each image with a lateral resolution of 0.5 cm^2^.

Attenuated total reflectance FTIR (ATR-FTIR, model is50 Thermo Scientific Nicolet FTIR spectrophotometer (Waltham, MA, USA, with a standard spectral range of 7800–350 cm^−1^ and optical resolution of no less than 0.09 cm^−1^) was used to determine the presence of characteristic functional groups of albumen. The final IR spectrum of the object examined was averaged from at least 16 runs.

### 4.3. Objects—Albumen Vintage Photographs

Together with the invention of photography in the nineteenth century, there began to appear a variety of booklets and journals containing photographs, which began to be called photograph albums. As a result, a whole range of different types of photographic albums began to appear, which—in contrast to existing systems—were divided into groups differentiated by form and function. The first and oldest group were the so-called artificial photo albums (the term was borrowed from the “factice atlas”), which began to be created from 1839 and perhaps even earlier. Each ‘artificial’ photo album existed in a single, unique copy. Photographs were selected specifically to illustrate the phenomena described, and they were bound by a bookbinder on the commission of the owner: photographer, researcher, artist, or collector. The second group of albums identified comprised albums of photographic trifles, aphorisms, and cuttings, in other words, scrapbooks (French Bêtisier, German Spruchalbum). All of them were unique, and they began to be created in 1839 and used for the purpose of exercise books, notebooks, sketchbooks, and diaries, which were commercially available. The latter often had richly decorated canvas covers with deeply impressed gold decoration—most often made in a historicizing style. The third group of materials comprised a large number of published photographic albums (PPA). This could be divided into subgroups regarding the types of illustrations which they contained. There are published albums with original photographs, those with reproductions, and those that mix both originals and reproductions. Such albums owe their existence to the development of the negative–positive photographic process, which allowed a large number of prints to be made from the same negative. Albums of this kind began to be published in 1844, but those with original photos tipped in were mainly made before 1900. Many albums of this type were produced. In them, the photographs played a dominant role in relation to the text, as the content of the volume was conveyed mainly through the universal language of visual images. Next, the fourth group comprised photographic albums, such as books of devotion (Huldigungsadresse), thanksgiving (Glückwunschadresse), and gratulatory address books (Adressbuch). They originated from the custom known from earlier eras of making a formal and symbolic act of recognition and loyalty submitted to the monarch by his subjects. The fifth and rather exceptional group is the family photograph albums, the creation of which, as opposed to the albums in the previously mentioned groups, related solely to the flourishing of photographic cartes-de-visite and other subsequent types of photographs of fixed format, such as Cabinet (1864), Victoria (from 1870) Oblong/Promenade (1875), Boudoir (1875) and Imperial (from 1875) formats.

Albums of this type constitute a homogenous group in terms of their purpose, intended mainly for the photographs of a family. They are, however, diverse in terms of form. For this reason, we may differentiate the following subgroups from among them: classic family photo albums (CFPA)—often mimicking the appearance of richly decorated manuscripts and old printed books, and atypical family photo albums—for example, in leporello format, which is an album standing on an easel into which copies of music movements or clocks are incorporated. There are also special-purpose family photo albums. These usually comprised a volume created to celebrate a special event or anniversary and were made to special order, created by goldsmiths or the bookbinder, which had some kind of distinguishing marks indicating its origin [29].

Vintage albumen photographs constituting the research group come from the following albums:

- Karol Beyer “Views of Warsaw” (“Widoki Warszawy”), [MW AF 9773], (PPA 1);
- Karol Beyer “Views of Warsaw” (“Widoki Warszawy”), [MNW DI 105371 (1–24)], second copy-other edition, (PPA 2);
- Konrad Brandel “Views of Warsaw” (“Widoki Warszawy”), [MW AL 78], edition in brown canvas, (PPA 3);
- Konrad Brandel “Views of Warsaw” (“Widoki Warszawy”), [MW AL. 79], edition in red canvas, (PPA 4);
- Album, [MHK Fs 4452/IX/31], (CFPA 1);
- Album, [AGAD,Division III, nr IX], (CFPA 2);
- Album, [AGAD, Division III, nr V], (CFPA 3).

Table S1 summarizes all important information on the albumen photographs examined in this work. Two representative photographs are presented in Figure S5 (Supplementary Material).

## 5. Conclusions

The examination of the inorganic (by XRF) and organic (by FTIR) characteristics of vintage photographs by the Principal Component Analysis technique resulted in the formulation of theoretical models that can express the major components (Z) in terms of the relevant measured quantities used. According to the models derived, the two major components (Z1 and Z2) explain a considerable part of the total variability observed for the experimental data. The question arises as to whether these components were able to distinguish the photographic objects in terms of their authorship and time of creation. The search for these answers was demonstrated by a select group of photographs consisting of albumen prints from three Warsaw photographic ateliers: Karol Beyer, Konrad Brandel, and Jan Mieczkowski. The interpretation of the PCA results was a very difficult task and was based on many hypotheses and existing historical knowledge about these objects. The analysis presented in this work definitely allows one to distinguish some differences between vintage photo objects from the selected photographic ateliers.

The prepared reference albumen photo samples served as materials for testing the performance of the XRF technique employed for the analysis of the silver content in photographic objects. The empirical functional relationships between the content of photochemically reduced silver particles on the photographic paper and several physicochemical parameters (including the content of silver (I) absorbed by the photographic paper and the wavenumbers of the characteristic bands for albumen), which can be used for the prediction of the experimental conditions under which the investigated photographs were developed.

## Figures and Tables

**Table 1:** The characteristics of chemicals employed in this experimental work.

Chemical	Purity	Manufacturer	Role
Silver nitrate (AgNO_3_)	99.9%	Honeywell, Tokyo, Japan	Silver ions source, photosensitive medium
Sodium thiosulfate pentahydrate (Na_2_S_2_O_3_·5H_2_O)	99.5%	Sigma Aldrich, St. Louis, MI, USA	Complexing agent
Citric acid monohydrate (C_6_H_8_O_7_·H_2_O)	>99%	POCh Gliwice, Gliwice, Poland	Reducing agent
Ammonium chloride (NH_4_Cl)	>99%	POCh Gliwice, Gliwice, Poland	Precipitating agent
Aluminum potassium sulfate dodecahydrate pure (AlK(SO_4_)_2_·12H_2_O)	>99%	WarChem Sp.z.o.o., Zakręt, Poland	Antibacterial agent
Albumen	Unknown	Custom preparation	Base for silver ions
Gelatine-covered paper: Bovine gelatin	-	Photogelatine Type Restoration 1, (GMW Germany, Gelita, Eberbach, Germany)	Base for albumen, Photosensitive medium
Whatman blotting paper	-	Whatman blotting paper 1 (GE Healthcare Life Sciences WhatmanTM, GE Healthcare UK Limited, Glasgow, UK)	Base for albumen, Photosensitive medium
Ultrapure water	0.055 µS·cm^−1^ *	Milli-Q system	Solvent, Liquid used for washing process

* Water conductivity at 25 °C.

## Data Availability

Data are contained within the article and Supplementary Materials.

## Supplementary Materials

The following supporting information can be downloaded at: https://www.mdpi.com/article/10.3390/molecules29102170/s1, Table S1. Description of albumen vintage photographs; Figure S1: Representative samples of reference albumen photographs (a) treated with solutions of the selected AgNO_3_ concentration in a fixed bath and illuminated by UV radiation for 120 s and (b) exposed to UV light acting on paper (treated with 5% AgNO_3_ salt in a fixed bath) for various exposure times; Figure S2: Relations between the resulting XRF content of metallic silver particles (*x_Ag_* (XRF)), UV illumination time (*t*) and concentration of AgNO_3_ salt (*c_AgNO3_*) in a fixed bath. White dots represent AgNO_3_ concentration in the fixed bath equal to 1.5%, where the red color is used for *c_AgNO3_* = 2.5%, green for *c_AgNO3_* = 5%, and black for *c_AgNO3_* = 10%; Figure S3: Representative XRF spectra with spectral assignations recorded for the selected vintage photograph (B1). Spectrum (a) corresponds to the photograph and (b) the background paper; Figure S4: A representative IR spectrum recorded for the selected vintage photographs (BR11) marked with symbols for characteristic frequencies; Figure S5: Two representative research objects–albumen vintage photographs.
