# Supplementary material for: Studies on the Authorship of Albumen Vintage Photographs: A Combined Experimental and Chemometric Approach
Source: Molecules. 2024 May 7;29(10):2170. doi: 10.3390/molecules29102170 (PMC11124433; doi:10.3390/molecules29102170)
Supplement: Supplementary file 1 [file molecules-29-02170-s001.zip › molecules-2978023-supplementary.pdf]

Supplementary Material

Studies on the authorship of albumen vintage photographs. Combined experimental and chemometric approach

Monika Adamowska [1], Izabela Zając [3], Marek Grzegorz Sawicki [1] and Wojciech Hyk [1,2,*]

[1] Faculty of Chemistry, University of Warsaw, Pasteura 1, PL-02-093 Warsaw, Poland
[2] Faculty of Chemistry, Biological and Chemical Research Center, University of Warsaw, Żwirki i Wigury 101, PL-02-089 Warsaw, Poland
[3] Faculty of Conservation and Restoration of Works of Art, Academy of Fine Arts in Warsaw, Wybrzeże Kościuszkowskie 37, PL-00-379 Warsaw, Poland
***** Correspondence: wojhyk@chem.uw.edu.pl; Tel.: (48-22) 5526359

Table S1. Description of albumen vintage photographs [23].

No.	Photo	Format (cm x cm) * Paper suport / photo	Symbol	Author	From ** (signature)	Time	Album
1.	City view	51.5x40 / 24x18	B1	Karol Beyer	MW (AF 9773)	1858-1859	PPA 1
2.	City view	51.5x40 / 24x18	B2	Karol Beyer	MW (AF 9766)	1858-1859	PPA 1
3.	City view	51.5x40 / 24x18	B3	Karol Beyer	MNW (DI 105371 (1-24))	1858-1859	PPA 2
4.	City view	51.5x40 / 24x18	B4	Karol Beyer	MNW (DI 105371 (1-24))	1858-1859	PPA 2
5.	City view (Figure S1 A)	51.5x40 / 24x18	B5	Karol Beyer	MNW (DI 105371 (1-24))	1858-1859	PPA 2
6.	City view	51.5x40 / 24x18	B6	Karol Beyer	MNW (DI 105371 (1-24))	1858-1859	PPA 2
7.	City view	51.5x40 / 24x18	B7	Karol Beyer	MNW (DI 105371 (1-24))	1858-1859	PPA 2
8.	City view	51.5x40 / 24x18	B8	Karol Beyer	MNW (DI 105371 (1-24))	1858-1859	PPA 2
9.	Portrait no. 31	CV	B9	Karol Beyer	MHK (Fs 4452 / IX / 31)	1860s	CFPA 1
10.	Portrait no. 48	CV	B10	Karol Beyer	MHK (Fs 4452 / IX / 48)	1860s	CFPA 1
11.	City view	33x25 / 15.5x12	BR1	Konrad Brandel	MW (AL 78)	~1870	PPA 3
12.	City view	33x25 / 15.5x12	BR2	Konrad Brandel	MW (AL 78)	~1870	PPA 3
13.	City view	33x25 / 15.5x12	BR3	Konrad Brandel	MW (AL 78)	~1870	PPA 3
14.	City view	33x25 / 15.5x12	BR4	Konrad Brandel	MW (AL 78)	~1870	PPA 3
15.	City view	33x25 / 15.5x12	BR5	Konrad Brandel	MW (AL 78)	~1870	PPA 3
16.	City view	33x25 / 15.5x12	BR6	Konrad Brandel	MW (AL 79)	~1870	PPA 4
17.	City view	33x25 / 15.5x12	BR7	Konrad Brandel	MW (AL 79)	~1870	PPA 4
18.	City view	33x25 / 15.5x12	BR8	Konrad Brandel	MW (AL 79)	~1870	PPA 4

19.	City view	33x25 / 15.5x12	BR9	Konrad Brandel	MW (AL 79)	~1870	PPA 4
20.	City view	33x25 / 15.5x12	BR10	Konrad Brandel	MW (AL 79)	~1870	PPA 4
21.	Portrait no. 1 (Figure S1 B)	CV	M1	Jan Mieczkowski	AGAD (Division III, no. IX)	1864-1870	CFPA 2
22.	Portrait no. 2	CV	M2	Jan Mieczkowski	AGAD (Division III, no. IX)	1864-1870	CFPA 2
23.	Portrait no. 9	CV	M3	Jan Mieczkowski	AGAD (Division III, no. IX)	1864-1870	CFPA 2
24.	Portrait no. 27	CV	M4	Jan Mieczkowski	AGAD (Division III, no. IX)	1864-1870	CFPA 2
25.	Portrait no. 42	CV	M5	Jan Mieczkowski	AGAD (Division III, no. IX)	1864-1870	CFPA 2
26.	Portrait no. 43	CV	M6	Jan Mieczkowski	AGAD (Division III, no. IX)	1864-1870	CFPA 2
27.	Portrait no. 44	CV	M7	Jan Mieczkowski	AGAD (Division III, no. IX)	1864-1870	CFPA 2
28.	Portrait no. 50	CV	M8	Jan Mieczkowski	AGAD (Division III, no. IX)	1864-1870	CFPA 2
29.	Portrait no. 51	CV	M9	Jan Mieczkowski	AGAD (Division III, no. IX)	1864-1870	CFPA 2
30.	Portrait no. 63	CV	M10	Jan Mieczkowski	AGAD (Division III, no. IX)	1864-1870	CFPA 2
31.	Portrait no. 65	CV	M11	Jan Mieczkowski	AGAD (Division III, no. IX)	1864-1870	CFPA 2
32.	Portrait no. 66	CV	M12	Jan Mieczkowski	AGAD (Division III, no. IX)	1864-1870	CFPA 2
33.	Portrait no. 67	CV	M13	Jan Mieczkowski	AGAD (Division III, no. IX)	1864-1870	CFPA 2
34.	Portrait no. 86	CV	M14	Jan Mieczkowski	AGAD (Division III, no. IX)	1864-1870	CFPA 2
35.	Portrait no. 87	CV	M15	Jan Mieczkowski	AGAD (Division III, no. IX)	1864-1870	CFPA 2

36.	Portrait no. 8	CC	M16	Jan Mieczkowski	AGAD (Division III, no. V)	1890	CFPA 3
37.	Portrait no. 9	CC	M17	Jan Mieczkowski	AGAD (Division III, no. V)	1890	CFPA 3
38.	Portrait no. 11	CC	M18	Jan Mieczkowski	AGAD (Division III, no. V)	1890	CFPA 3
39.	Portrait no. 12	CC	M19	Jan Mieczkowski	AGAD (Division III, no. V)	1890	CFPA 3
40.	Portrait no. 13	CC	M20	Jan Mieczkowski	AGAD (Division III, no. V)	1890	CFPA 3
41.	Portrait no. 14	CC	M21	Jan Mieczkowski	AGAD (Division III, no. V)	1890	CFPA 3

* CV – cartes- de - visite; CC – cabinet card

** MHK – Historical Museum in Cracow; MNW – National Museum in Warsaw; MW – Museum of Warsaw;
AGAD – The Central Archives of Historical Records in Warsaw

PPA – Published Photograhic Albums

CFPA – Classical Family Photoalbums

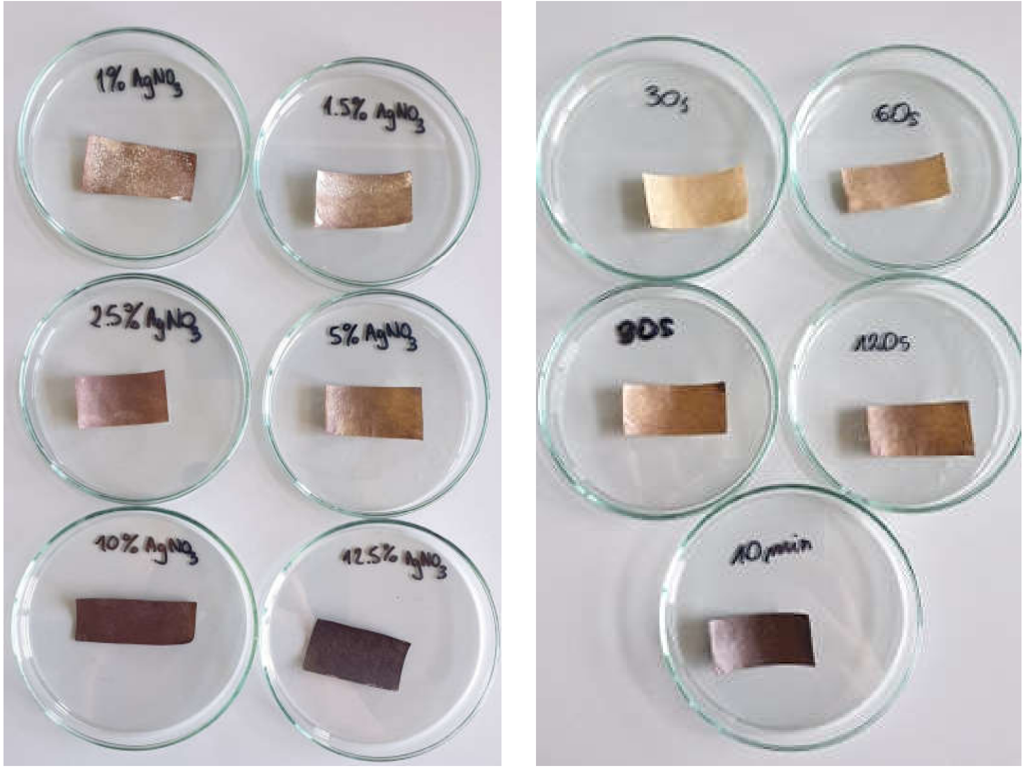

Figure S1. Representative samples of reference albumen photographs: (a) treated with solutions of the selected $AgNO_3$ concentration in the fix bath and illuminated by UV radiation by 120 s and (b) exposed to UV light acting on paper (treated with 5% $AgNO_3$ salt in fix bath) for various exposure times.

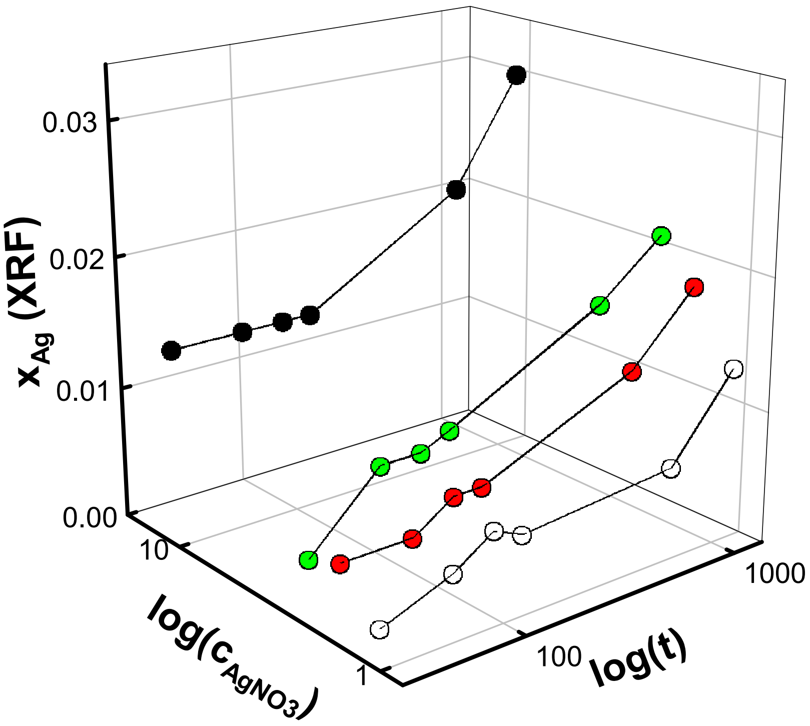

Figure S2. Relations between the resulting XRF content of metallic silver particles (x_{Ag} (XRF)), UV illumination time (t) and concentration of $AgNO_3$ salt (c_{AgNO3}) in the fix bath. White dots represent $AgNO_3$ concentration in the fix bath equal to 1.5 %, red color was used for c_{AgNO3} = 2.5 %, green for c_{AgNO3} = 5 % and black for c_{AgNO3} = 10 %.

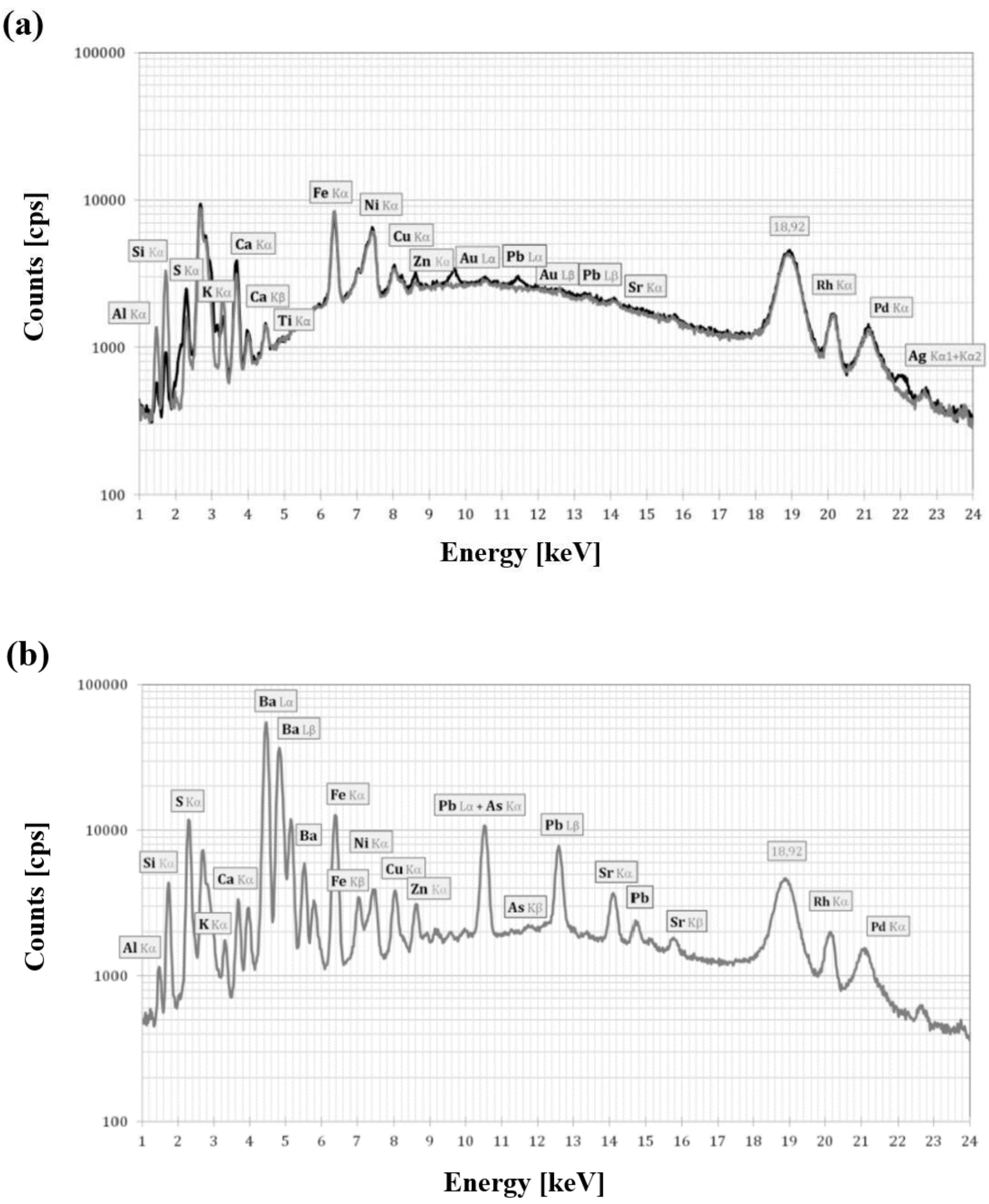

Figure S3. Representative XRF spectra with spectral assignations recorded for the selected vintage photograph (B1). Spectrum a) corresponds to the photograph and b) – to background paper.

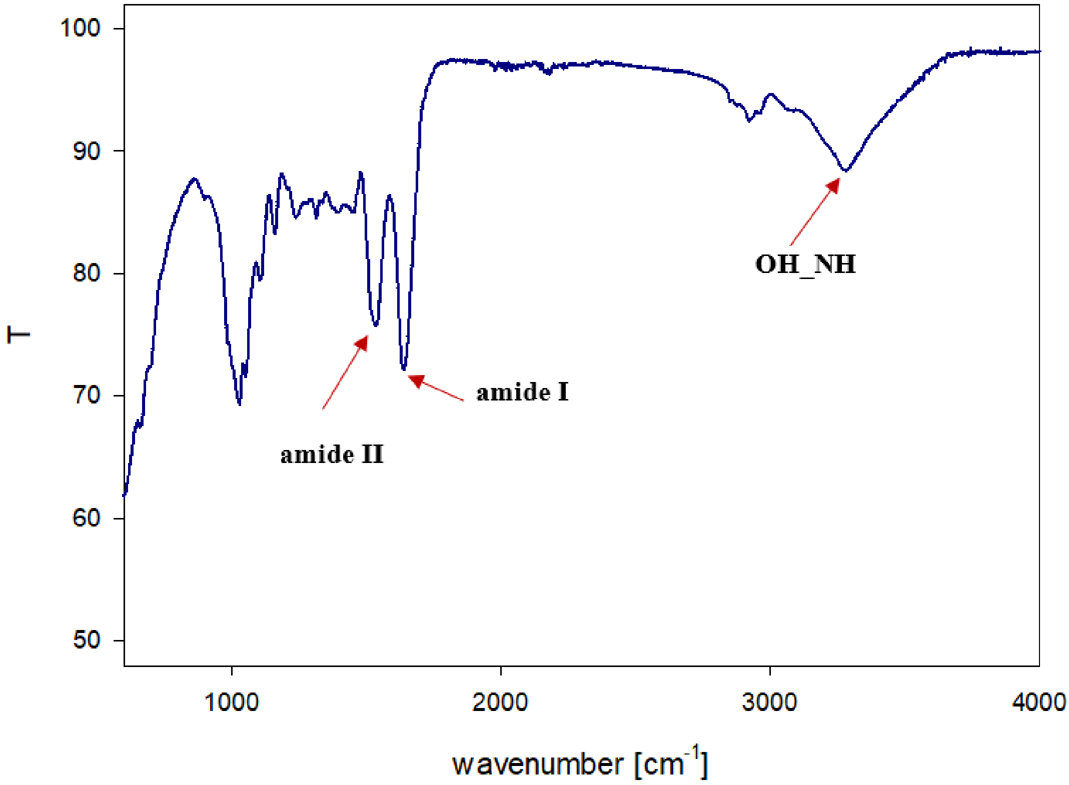

Figure S4. A representative IR spectrum recorded for the selected vintage photograph (BR11) marked with symbols for characteristics frequencies.

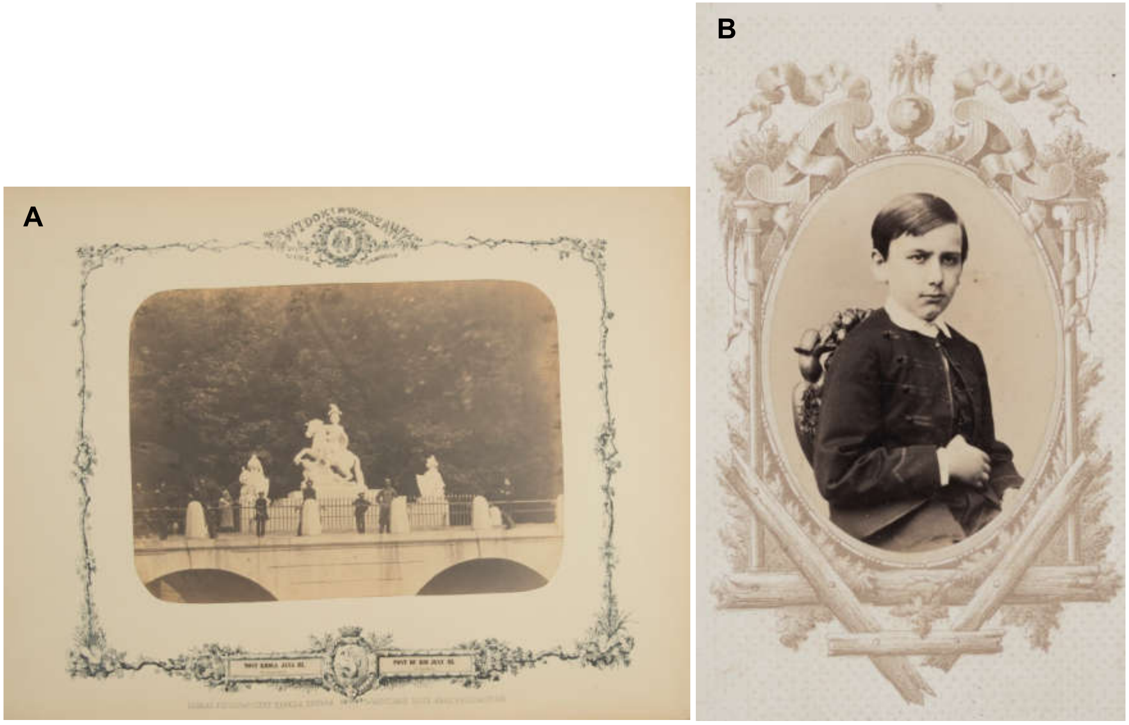

Figure S5. Two representative research objects – albumen vintage photographs.
